# Adaptive Particle Filter for Nonparametric Estimation with Measurement Uncertainty in Wireless Sensor Networks

**DOI:** 10.3390/s16060786

**Published:** 2016-05-30

**Authors:** Xiaofan Li, Yubin Zhao, Sha Zhang, Xiaopeng Fan

**Affiliations:** 1 The State Monitoring Center and Testing Center, Beijing 100037, China; lixiaofan@srtc.org.cn (X.L.); zhangsha@srtc.org.cn (S.Z.); 2 Shenzhen Institute of Radio Testing & Tech., Shenzhen 518000, China; 3 Shenzhen Institutes of Advanced Technology, Chinese Academy of Sciences, Shenzhen 518055, China; xp.fan@siat.ac.cn

**Keywords:** particle filter, nonparametric estimation, indoor positioning, wireless sensor networks, Kullback–Leibler divergence, dynamic Gaussian model

## Abstract

Particle filters (PFs) are widely used for nonlinear signal processing in wireless sensor networks (WSNs). However, the measurement uncertainty makes the WSN observations unreliable to the actual case and also degrades the estimation accuracy of the PFs. In addition to the algorithm design, few works focus on improving the likelihood calculation method, since it can be pre-assumed by a given distribution model. In this paper, we propose a novel PF method, which is based on a new likelihood fusion method for WSNs and can further improve the estimation performance. We firstly use a dynamic Gaussian model to describe the nonparametric features of the measurement uncertainty. Then, we propose a likelihood adaptation method that employs the prior information and a belief factor to reduce the measurement noise. The optimal belief factor is attained by deriving the minimum Kullback–Leibler divergence. The likelihood adaptation method can be integrated into any PFs, and we use our method to develop three versions of adaptive PFs for a target tracking system using wireless sensor network. The simulation and experimental results demonstrate that our likelihood adaptation method has greatly improved the estimation performance of PFs in a high noise environment. In addition, the adaptive PFs are highly adaptable to the environment without imposing computational complexity.

## 1. Introduction

With the advancement of wireless sensor network (WSNs) and RFID technologies, the demands for indoor location information have promoted the extensive study of indoor localization methods over the past few years [1]. There are increasing requirements of location-based services (LBS) in practical applications, such as indoor navigation systems, location-based social networks, indoor robot and healthcare [2]. The reliability of the location accuracy is a major concern when providing the location information. Additionally, the target position can be obtained via range-based measurement, such as time-of-arrival (TOA), time-difference-of-arrival (TDOA) and received signal strength (RSS), by using WSNs or RFID. In a dynamic high noise environment, the multi-path and non-line-of-sight (NLOS) effects cause high interference during the signal transmission, and the range measurement contains high error. In this case, the PF is a promising estimation method in the nonlinear non-Gaussian environment due to its high accuracy and is widely applied in the indoor localization systems.

A particle filter (PF), which is a typical Bayesian estimation method, has drawn much attention in the fields of state estimation and signal processing for many years [3]. Many applications employ the PF to process the collected data, such as target tracking [4,5] and localization systems [6]. Based on Bayes’ theorem, PFs generate random samples, which are called particles with associated weights to represent the posterior PDF of the state. The associate weights are proportional to the prior PDF and the likelihoods and updated recursively.

However, the estimation accuracy of the PF can also be degraded due to the range measurement uncertainty. A traditional way is to generate high quality particles, since the PF is introduced to the localization problem [7]. The bootstrap particle filter (BPF) is applied to generate and re-sample the particles during the recursive position estimation [6]. Farahmand *et al.* proposed a set-membership constraint particle filter (CPF) to select particles within the constrains [8,9]. Kwok *et al.* introduce an adaptable sampling method based on the Kullback–Leibler divergence (KLD) [10]. The Gaussian particle filter (GPF) generates the particles according to the Gaussian distributions and also approximates the estimated distribution as the Gaussian distributions [11]. The accuracy of the GPF is widely evaluated in RFID and WSN-based indoor tracking [12,13]. Ristic *et al.* proposed probability hypothesis density (PHD) and cardinalised PHD (CPHD) filters for target tracking [14]. The other solution is to use an efficient wireless model to overcome the NLOS and multipath effects. The NLOS channel is believed to introduce additional bias error rather than the LOS ranging error. Thus, NLOS path identification and bias reduction methods are widely applied in the PF designs. Jung *et al.* identify the NLOS path for the TOA ranging and use PFs to estimate the target according to the biased error model [15]. Youssef *et al.* provide the biased model of the RSS NLOS ranging error [16]. In addition, the map information can also help to indicate the NLOS path [17]. The problem is that the NLOS error cannot be simply modeled as an LOS error model plus a bias. A typical error model is required for the NLOS channel. The Markov state-space model for NLOS error is usually introduced into the PF-based indoor localization. Nicoli *et al.* developed a recursive Bayesian estimator that combines the Markov transition model and the NLOS propagation in the indoor environment [18]. Wang *et al.* introduce the NLOS error model to the Markov model of the PF based on 802.15.4a [19]. Papa *et al.* proposed adaptive Bayesian methods to track the target using a sensor network [20]. In addition, more information is employed to reduce the measurement error. The hybrid information of TOA and RSS is exploited for likelihood calculation in [21]. Further, the combination of TOA/RSS with NLOS and the multipath mitigation method is introduced in [22]. Bargshady *et al.* also use the PF to process the hybrid WiFi and ultra wideband (UWB) measurements to reduce the NLOS effect [23].

The PF achieves high estimation accuracy by using the prior probability and likelihood based on the Bayesian method. The likelihood relies on the measurement error distribution. However, there is no exact distribution to represent the measurement noise in the complicated indoor environment. Even if the distribution fitting or wireless propagation modeling methods can derive the model parameters, these parameters should be reset when the environment is changed, such as an infrastructure change or the target moves to another area. Thus, the indoor measurement noise shows nonparametric features [24], which requires that the PF is robust to the unknown error models. In this paper, we propose a novel method to design the PF in order to achieve more accurate estimation in a complicated environment. We firstly analyze the measurement uncertainty for the likelihood function (LF) in the PF. The LF, which relies on the measurement and noise distribution, has a large proportion in determining the particle weights. Since the error model is unknown to the LF calculation, the posterior PDF of the state estimation is unreliable. We divide the measurement error model into two parts: the pre-assumed distribution and the unknown model, and use a dynamic Gaussian model (DGM) to comprehensively describe both parts [25]. The DGM assumes that the real-time measurement error follows the pre-assumed distribution, which is dynamically deviated by the instantaneous unknown error. Then, the likelihood function (LF) in the PF, which relies on the measurement function and noise distribution, is calculated based on the DGM.

Our goal is to reduce the instantaneous unknown noise in LF according to our analysis, and the major contributions of our work are four-fold: (1) We propose a likelihood adaptation method, which is based on the DGM. The method combines the prior information and a tuning parameter to reduce the instantaneous unknown error: the prior information is the predicted measurement based on the predicted state; and the belief factor θ is the tuning parameter, which adapts the likelihood function to a more accurate one. By tuning θ, the impact of unknown error for likelihood calculation is reduced; (2) In order to obtain the optimal performance, we use the Kullback–Leibler divergence (KLD), which is an efficient metric to compare two distributions, to derive the optimal θ. The optimal θ can achieve the minimum KLD and attain the lowest estimation error of the PF; (3) We formulate the optimal performance of the adaptive method by using the Cramér–Rao lower bound (CRLB) analysis. The analytical results indicate that our method outperforms the conventional PF for the nonparametric measurement error models; (4) Three versions of PFs are improved based on our adaptation method, which are the BPF, the GPF and the CPF. The improved PFs are evaluated in the simulations and the real-world WSN indoor localization experiments. The results demonstrate that the proposed algorithms effectively reduce the estimation error and have robust performance in a high noisy wireless environment.

The rest of the paper is organized as follows: Section 2 analyzes the measurement uncertainty effect of the likelihood function in the PF algorithms. Section 3 describes a dynamic Gaussian modeling method for the uncertainty and outlier measurement errors. Section 4 proposes the likelihood adaptation method, and the adaptive PFs are designed in Section 5. The theoretical analysis based on CRLB is described in Section 6. Section 7 presents the simulation and real experiment results of adaptive PFs in the indoor localization systems. Finally, Section 8 concludes this paper.

## 2. Particle Filter Estimation Model

### 2.1. State-Space Model

In general, the state-space model is applied to describe the nonlinear estimation problem based on the hidden Markov process. The estimated state vector $x_{t}$ is evolved from the current observation $z_{t}$ and the previous state $x_{t-1}$, which are defined as the prediction equation and the update equation. The prediction equation is:

(1) $$x_{t}=f_{t}\left(x_{t-1}\right)+q_{t}$$

where $f_{t}\left(\right)$ is the prediction function, $x_{t-1}$ is the previous state and $q_{t}$ is the prediction noise, which implies the prediction error of $x_{t}$. Here, we assume that $q_{t}$ is additive to the prediction.

At time *t*, the system obtains a noisy measurement vector $z_{t}$. The relationship between $x_{t}$ and $z_{t}$ follows:

(2) $$z_{t}=h_{t}\left(x_{t}\right)+v_{t}$$

where $h_{t}\left(\right)$ is the measurement function and $v_{t}$ is the measurement noise at time *t*. In this paper, we employ the TOA measurement for range information. The TOA is a simple and straightforward range measurement method for indoor localization. The distance is proportional to the transmission delay, and the noise comes from the additive delay, which is introduced by the multi-path, NLOS and unsynchronized distributed clock within the sensors. The TOA is generally formulated as:

(3) $$\tau =\frac{1}{c}\left|\right|x_{t}-a\left|\right|$$

where *τ* is the transmission delay, *c* is the microwave speed, $a={\left[s_{x},s_{y}\right]}^{T}$ is the position vector of the anchor and $\left|\right|x_{t}-a\left|\right|$ indicates the distance between the target and the anchor. Considering the noise, the range measurement for each anchor *j* can be formulated as:

(4) $$z_{t}^{j}=h_{t}^{j}\left(x_{t}\right)+v_{t}^{j}=\left|\right|x_{t}-a^{j}\left|\right|+v_{t}^{j}$$

where $x_{t}=\left[p_{x}^{t}\;p_{y}^{t}\right]$ is the target position and $a^{j}={\left[s_{x}^{j},s_{y}^{j}\right]}^{T}$ is the position of anchor *j*. For a 2D positioning system, the distance measurement function can be expressed as:

(5) $$h_{t}^{j}\left(x_{t}\right)=\left|\right|x_{t}-a^{j}\left|\right|=\sqrt{{\left(p_{x}^{t}-s_{x}^{j}\right)}^{2}+{\left(p_{y}^{t}-s_{y}^{j}\right)}^{2}}$$

then, $z_{t}=\left[z_{t}^{1}\;\ldots \;z_{t}^{j}\;\ldots \;z_{t}^{N}\right]$, where *N* indicates the number of anchors. Accordingly, $p(z_{t}|x_{t})$ is defined as the measurement likelihood for $z_{t}$, which is essential to the whole estimation.

### 2.2. Particle Filter

PF consists of three parts: the first part is to generate a set of state samples, which are called particles; the second part is to calculate the associated weight for each particle; the final part is to re-sample the particles and to eliminate the unimportant particles. The estimated state is obtained by calculating the weighted average values of the particles. PF first generates a particle set ${\left\{x_{t}^{i},w_{t}^{i}\right\}}_{i=1}^{N_{s}}$ according to the importance density function $I_{s}\left(x_{t}^{i}\right)$, where ${\left\{x_{t}^{i}\right\}}_{i=1}^{N_{s}}$ is the sample set with associate weights ${\left\{w_{t}^{i}\right\}}_{i=1}^{N_{s}}$; *i* and $N_{s}$ denote the particle number and the total number, respectively. The purpose of using $I_{s}\left(x_{t}^{i}\right)$ is to generate high quality particles and then to attain the accurate estimation. Since the particles represent the possible state in the state space, it is necessary to sample a finite particles with high probability that can represent the state due to the limited computational overhead. Thus, the high quality particles are the samples with a high probability generated and calculated from $I_{s}\left(x_{t}^{i}\right)$. Based on $I_{s}\left(x_{t}^{i}\right)$, most particles are drawn only in the interested area, and a few particles are sampled on the other places. Thus, the posterior PDF $p(x_{t}|z_{t})$ of state $x_{t}$ can be approximated by using the delta function:

(6) $$p\left(x_{t}|z_{t}\right)\approx \sum_{i=1}^{N_{s}}w_{t}^{i}\delta \left(x_{t}-x_{t}^{i}\right)$$

For the Bayesian estimation in a non-Gaussian environment, it hardly provides the analytical formulation. Thus, the approximation is used with the set of particles, which is the main advantage of the PF [26]. Additionally, the weight of each particle can be calculated based on the Bayes rule [27]:

(7) $$w_{t}^{i}\propto w_{t-1}^{i}\frac{p\left(z_{t}|x_{t}^{i}\right)p\left(x_{t}^{i}|x_{t-1}^{i}\right)}{I_{s}\left(x_{t}^{i}|x_{0:t-1}^{i},z_{t}\right)}$$

where $p(x_{t}^{i}|x_{t-1}^{i})$ is the transition probability for particle $x_{t}^{i}$ and $p(z_{t}|x_{t}^{i})$ is the measurement likelihood of $z_{t}$ for $x_{t}^{i}$. The importance density function $I_{s}\left(x_{t}^{i}\right)=I_{s}\left(x_{t}^{i}|x_{0:t-1}^{i},z_{t}\right)$ draws new samples ${\left\{x_{t}^{i}\right\}}_{i=1}^{N_{s}}$, which evolves from the previous samples ${\left\{x_{t-1}^{i}\right\}}_{i=1}^{N_{s}}$, and 0:t−1 denotes the time sequence from the initial state to the previous state.

The PF estimation relies on the high quality particles and associated weight calculations. The weight is obtained via Bayes’ rule, which considers the prior probability and the measurement likelihood. The likelihood relates to the measurement and the noise distributions. A proper error distribution can lead to an accurate estimation not only for the PF, but also for other probability-based algorithms, e.g., maximum likelihood (ML) and maximum *a posterior* (MAP) algorithms.

### 2.3. The Measurement Uncertainty

In PFs, the measurement likelihood for each particle is calculated as [26]:

(8) $$p\left(z_{t}|x_{t}^{i}\right)=p\left(h_{t}\left(x_{t}\right)-z_{t}^{i}+v_{t}\right)$$

where $z_{t}^{i}=h_{t}\left(x_{t}^{i}\right)$ and p() is the PDF of measurement noise $v_{t}$ with variables of $z_{t}^{i}$. The additive measurement noise is assumed to be zero mean and follows a Gaussian distribution. However, the statistical results indicate that the noise is not zero mean due to heavy outlier values [28]. Thus, other PDFs are applied for measurement noise modeling, e.g., exponential, Rayleigh, Weibull or Gamma distribution [29]. If such a PDF is known to the system, the PF can obtain an accurate estimation, which approaches the optimal estimation with an increasing number of particles, as CRLB indicates. However, the PDF is not always known or precise for real applications. In real applications, the measurement error is modeled by statistical methods based on the sample dataset. On the one hand, the parameters of the error model can be changed if the sample dataset is changed. On the other hand, the outlier values can deviate from the error distribution, which makes the error model inaccurate for the PF estimation.

**Figure 1:**
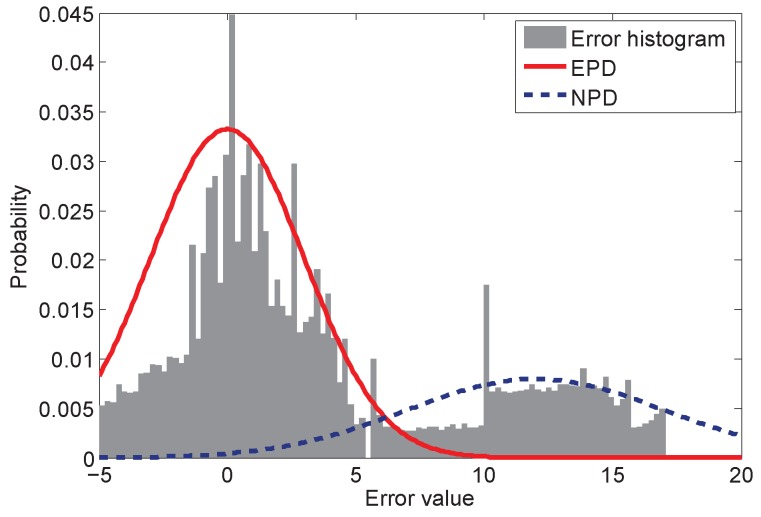
Measurement error histograms and the dynamic Gaussian model for measurement uncertainty.

For instance, we construct an experiment to collect all of the ranging measurement noise throughout the whole building. During such an experiment, we employ a robot moving automatically through every possible position of the building, including the hallway, the office room and classroom in a building at Freie University Berlin. Then, all of the ranging errors are collected, and the histogram is constructed. The error data can be attained freely via the website of [30]. The large positive noise, which may contribute a heavy tail for a histogram, is shown in Figure 1. In Figure 1, the histogram can be divided into two parts: the left part is similar to a Gaussian distribution, for which the mean and variance can be calculated; the right part is a kind of heavy tail, which represents the outlier values. To describe the histogram, researchers use an arbitrary assumed error model or a combined model to approach the real application. However, this makes the estimation algorithms sensitive to the environment. If the environment is changed, the model should be adapted accordingly; otherwise, it can lead to the wrong estimation.

## 3. Dynamic Gaussian Model

To address the measurement uncertainty problem and to make the PF robust to environmental change, we firstly introduce a dynamic Gaussian model (DGM) to the likelihood calculation. The main idea of the DGM is to classify the measurement error distribution into two parts: the first part is the expected parametric distribution (EPD), which is a pre-assumed distribution and known to the system. The EPD is obtained based on the knowledge or experiences of the system design, and the parameters can be attained via the system model or some pre-assumptions. The most popular EPD is the normal distribution. The second part is the non-parametric distribution (NPD), which is unknown to the system and for which it is hard to get the parameters. The conventional PFs only use EPD as the pre-assumed distribution model for estimation. The NPD is the compensation for the EPD when the PFs are in the dynamic environment without the knowledge of the noise model. The histogram of the EPD and the NPD is depicted in Figure 1. In Figure 1, the error value between −5 and 5 can mainly be modeled as the normal distribution, which is the EPD and denoted by the solid curve. For the outlier values, another Gaussian distribution NPD attempts to cover such values, which is depicted by the dashed curve.

The DGM is the drifted EPD, which is deviated by the instantaneous NPD value. Here, we use $v_{t}=w_{t}+n_{t}$, where $w_{t}$ is the EPD error and $n_{t}$ is the NPD error. Then, $v_{t}$ follows the EPD, but deviated by $n_{t}$. The DGM is different from the Gaussian mixture model. The Gaussian mixture model uses different Gaussian distributions to fit the non-Gaussian distributions. The results are still a static distribution model. However, DGM is dynamic and does not attempt to fit the error histogram, because the NPD part is not an exact model to fit the outlier values. It is rather a fuzzy function to cover such values. In addition, the Gaussian mixture model is a parametric model for the entirety of the error samples, and the DGM only contains a single instantaneous value of the NPD instead of the entirety of the data samples. In general, if $n_{t}=0$, $v_{t}$ follows the EPD. However, for a typical measurement, $v_{t}$ consists of a drift value $w_{t}$ that deviates from the pre-assumed normal distribution to a certain distance. Then, the EPD should be adapted dynamically according to the instantaneous value of the NPD.

**Figure 2:**
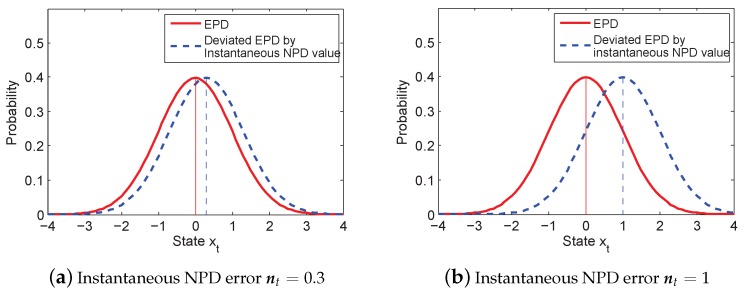
Example: the two likelihood functions. The solid curve represents the expected parametric distribution (EPD), and the dashed curve represents the deviated likelihood function by the instantaneous non-parametric distribution (NPD) value.

The DGM for a typical NPD error is depicted in Figure 2. In Figure 2, the solid curve represents the EPD, which is a zero mean normal distribution. When the instantaneous value of the NPD is introduced into the measurement noise, the EPD is deviated. Then, we obtain the likelihood calculation:

(9) $$p\left(z_{t}|x_{t}^{i}\right)=p\left(h_{t}\left(x_{t}\right)-z_{t}^{i}+w_{t}+n_{t}\right)$$

where an unpredictable instantaneous noise $n_{t}$ is introduced into the likelihood function.

Equation (9) is illustrated as the dashed curve in Figure 2, which is a biased non-zero mean Gaussian distribution. It is deviated from the original assumption due to considering the instantaneous value, $n_{t}$. If $n_{t}$ is zero, the likelihood function $p_{A}\left(z_{t}|x_{t}^{i}\right)$ is the exact EPD of measurement noise:

(10) $$p_{A}\left(z_{t}|x_{t}^{i}\right)=p\left(h_{t}\left(x_{t}\right)-z_{t}^{i}+w_{t}\right)$$

where $p_{A}$ represents the actual assumed probability; then, we would have optimal filtering with the increasing number of particles. However, in most real cases, $n_{t}$ is not zero, and $w_{t}$ is deviated by $n_{t}$ in Equation (10), which leads to inaccurate estimation. When $n_{t}$ becomes larger, the gap between the two curves is increasing, as shown in Figure 2b, which degrades the estimation accuracy significantly. Therefore, our goal is to develop an adaptation method to mitigate $n_{t}$ and approach the likelihood calculation of the exact assumed distribution.

## 4. Likelihood Adaptation

**Figure 3:**
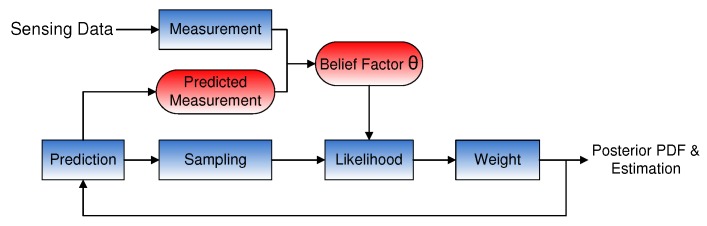
The architecture of the particle filter integrated with the adaptive likelihood method. The common particle filter does not contain the prior measurement and the belief factor.

We attempt to reduce the impact of the NPD and calculate the LF $p(z_{t}|x_{t}^{i})$ based on the DGM in order to improve the estimation accuracy. Our adaptation method consists of two steps: the first step is to obtain a predicted measurement ${\hat{z}}_{t}$ according to the previous state; the second step is to adapt the LF based on ${\hat{z}}_{t}$ and a belief factor θ, which is a tuning parameter for adaptation. The structure of the adaptive PF, which integrates with the predicted measurement and θ, is illustrated in Figure 3. In the original PF, the LF is determined by the EPD, whereas in our algorithm, the likelihood calculation is based on the DGM. Belief factor θ is used to adapt the value between ${\hat{z}}_{t}$ and $z_{t}$.

### 4.1. Predicted Measurement

The predicted measurement is derived based on the prediction state and is the reference for the real measurement. The calculation steps are as follows: ${\hat{x}}_{t}$ denotes the prediction value of $x_{t}$:

(11) $${\hat{x}}_{t}=f_{t}\left(x_{t-1}\right)$$

where $x_{t-1}$ is the estimation at previous time t−1. When considering the processing noise $q_{t}$, we denote ${\hat{x}}_{t}$ as:

(12) $${\hat{x}}_{t}=x_{t}+q_{t}$$

where $q_{t}$ is assumed to be the additive noise and follows normal distribution $q_{t}∼N\left(0,Q_{t}\right)$; $Q_{t}$ is the covariance at time *t*. Then, we obtain a predicted measurement for: sensors:

(13) $${\hat{z}}_{t}=h_{t}\left({\hat{x}}_{t}\right)=h_{t}\left(x_{t}+q_{t}\right)$$

in which ${\hat{z}}_{t}$ indicates the prediction of measurement derived from ${\hat{x}}_{t}$. The predicted measurement ${\hat{z}}_{t}$ is not the actual measurement, but is used as prior information for measurement likelihood adaptation.

### 4.2. Belief Factor and Measurement Adaptation

Belief factor θ is the tuning parameter for ${\hat{z}}_{t}$, and it is used to adapt the measurement $z_{t}$ to reduce $n_{t}$. Note that our goal is to achieve Equation (10). Since Equation (10) can not be achieved directly, we use ${\hat{z}}_{t}$ and $z_{t}$ to approach Equation (10) with θ. Then, the adaptive likelihood function $p_{AL}\left(z_{t}|x_{t}^{i}\right)$ is constructed as:

(14) $$\begin{array}{c} p_{AL}\left(z_{t}|x_{t}^{i}\right)=p\left(\theta ⊙{\hat{z}}_{t}+\left(1-\theta \right)⊙z_{t}-z_{t}^{i}\right) \end{array}$$

where $p_{AL}\left(\right)$ indicates the adaptive likelihood, $z_{t}$ is the measurement vector, ${\hat{z}}_{t}$ is the predicted measurement and $z_{t}^{i}=h_{t}\left(x_{t}^{i}\right)$. The notation ⊙ in Equation (14) is the Hadamard operator, which is used to expressed as $\theta ⊙{\hat{z}}_{t}={\left[\theta_{t}^{1}{\hat{z}}_{t}^{1}\ldots \theta_{t}^{j}{\hat{z}}_{t}^{j}\ldots \theta_{t}^{N}{\hat{z}}_{t}^{N}\right]}^{T}$, and 1 is the vector of ones whose size corresponds to that of θ. Note that θ≺1, which should be interpreted as the elements in 1−θ, are non-negative. The belief factor θ indicates how much trust $p_{AL}\left(z_{t}|x_{t}^{i}\right)$ assigns to the ${\hat{z}}_{t}$. If θ=0, $p_{AL}\left(z_{t}|x_{t}^{i}\right)=p\left(z_{t}-z_{t}^{i}\right)=p\left(z_{t}|x_{t}^{i}\right)$, which equals the measurement likelihood; whereas when θ=1, $p_{AL}\left(z_{t}|x_{t}^{i}\right)=p\left({\hat{z}}_{t}-z_{t}^{i}\right)$, which only trusts the predicted measurement.

Here, two problems remains: (1) with introducing θ<1, the measurement error in $z_{t}$ is suppressed, but the error of ${\hat{z}}_{t}$ is involved, which raises a question: in what condition can our method reduce $n_{t}$? (2) If our method can reduce this effect, a proper θ is required. Therefore, is there an optimal θ that can achieve the best performance?

### 4.3. Optimal Belief Factor and Likelihood Estimation

Since we intend to compare our adapted DGM to the EPD, the effective evaluation method is KLD [31]. KLD, which also is denoted as relative entropy, quantifies the difference between two distributions. If $p_{1}\left(x\right)$ and $p_{2}\left(x\right)$ indicate two different distributions, KLD is formulated as:

(15) $$D_{KL}(p_{1}\left|\right|p_{2})=\int p_{1}\left(x\right)\log\frac{p_{1}\left(x\right)}{p_{2}\left(x\right)}dx=E_{p_{1}}\left[\log\frac{p_{1}\left(x\right)}{p_{2}\left(x\right)}\right]$$

where $D_{KL}(p_{1}\left|\right|p_{2})$ denotes the KLD. The KLD is a non-negative distance between two different distributions, which is $D_{KL}(p_{1}\left|\right|p_{2})\geq 0$. It can be shown that $D_{KL}(p_{1}\left|\right|p_{2})=0\Leftrightarrow p_{1}\left(x\right)=p_{2}\left(x\right)$. Additionally, small $D_{KL}(p_{1}\left|\right|p_{2})$ indicates that $p_{1}\left(x\right)$ is similar to $p_{2}\left(x\right)$. In addition, KLD is a convex function [32].

We employ the KLD as a metric to find optimal θ, which minimizes the distance between $p_{A}\left(z_{t}|x_{t}\right)$ and $p_{AL}\left(z_{t}|x_{t}\right)$. Here, we use $p_{A}\left(z_{t}|x_{t}\right)$ as the objective distribution and employ $p_{AL}\left(z_{t}|x_{t}\right)$ as the tuning distribution with parameter θ. Then, the KLD function is constructed as:

(16) $$\begin{array}{cc} D_{KL}(p_{A}\left|\right|p_{AL}) & =\int p_{A}\left(z_{t}|x_{t}\right)\log\frac{p_{A}\left(z_{t}|x_{t}\right)}{p_{AL}\left(z_{t}|x_{t}\right)}dz_{t}^{i}\\ & =\int \pi_{v}\left(h_{t}\left(x_{t}\right)-z_{t}^{i}\right)\log\frac{\pi_{v}\left(h_{t}\left(x_{t}\right)-z_{t}^{i}\right)}{\pi_{v}\left(\theta ⊙{\hat{z}}_{t}+\left(1-\theta \right)⊙z_{t}-z_{t}^{i}\right)}dz_{t}^{i} \end{array}$$

Then, optimal θ is attained with minimum $D_{KL}(p_{A}\left|\right|p_{AL})$:

(17) $$\theta =\arg\min D_{KL}(p_{A}\left|\right|p_{AL})$$

If $p_{A}\left(\right)$ and $p_{AL}\left(\right)$ are based on the same Gaussian distribution function, then Equation (16) is expressed as [33]:

(18) $$D_{KL}(p_{A}\left|\right|p_{AL})=\frac{\left|\right|h_{t}\left(x_{t}\right)-\left[\theta ⊙{\hat{z}}_{t}+\left(1-\theta \right)⊙z_{t}\right]{\left|\right|}^{2}}{2R_{t}}$$

where $R_{t}$ is the covariance of $v_{t}$. Since $R_{t}$ is independent of θ, the objective function is simplified as:

(19) $$\begin{array}{c} \theta =\arg\min\;||h_{t}\left(x_{t}\right)-\left[\theta ⊙{\hat{z}}_{t}+\left(1-\theta \right)⊙z_{t}\right]{\left|\right|}^{2} \end{array}$$

which turns out to be a least-squares approximation problem [34]. Since ${\hat{z}}_{t}$ is the nonlinear functions of the prediction noise $q_{t}$ according to Equation (13), it is difficult to obtain an analytical optimal result. Besides, the computational complexity is increased accordingly to obtain an optimal solution. Thus, linearizing the object function is preferred. We use first order Taylor series expansion at $x_{t}$ to linearize Equation (13):

(20) $${\hat{z}}_{t}\approx h_{t}\left(x_{t}\right)+\frac{\partial h_{t}\left(x_{t}\right)}{\partial x_{t}}q_{t}$$

where $\partial h_{t}\left(x_{t}\right)/\partial x_{t}$ is the partial differential of $h_{t}\left(x_{t}\right)$ with respect to $x_{t}$. Additionally, substitute Equation (20) and Equations (2) into (19); we obtain:

(21) $$\left|\right|h_{t}\left(x_{t}\right)-\left[\theta ⊙{\hat{z}}_{t}+\left(1-\theta \right)⊙z_{t}\right]{\left|\right|}^{2}\approx ||\theta ⊙\frac{\partial h_{t}\left(x_{t}\right)}{\partial x_{t}}q_{t}+\left(1-\theta \right)⊙v_{t}{\left|\right|}^{2}$$

Therefore, the problem is converted into a linear optimization problem, which is solvable analytically by expressing the objective as the convex quadratic function [34]:

(22) $$F_{t}\left(\theta \right)=\theta \frac{\partial h_{t}\left(x_{t}\right)}{\partial x_{t}}Q_{t}{\left[\frac{\partial h_{t}\left(x_{t}\right)}{\partial x_{t}}\right]}^{T}\theta^{T}+\left[1-\theta \right]R_{t}{\left[1-\theta \right]}^{T}$$

where $Q_{t}$ is the covariance of $q_{t}$ and $R_{t}$ is the covariance of $n_{t}$ based on the assumed NPD model.

Then, the optimal θ can be obtained if and only if:

(23) $$\frac{\partial F_{t}\left(\theta \right)}{\partial \theta }=2\theta \frac{\partial h_{t}\left(x_{t}\right)}{\partial x_{t}}Q_{t}{\left[\frac{\partial h_{t}\left(x_{t}\right)}{\partial x_{t}}\right]}^{T}-2R_{t}+2\theta R_{t}=0$$

Then, the unique θ is derived:

(24) $$\theta =\frac{R_{t}}{\frac{\partial h_{t}\left(x_{t}\right)}{\partial x_{t}}Q_{t}{\left[\frac{\partial h_{t}\left(x_{t}\right)}{\partial x_{t}}\right]}^{T}+R_{t}}$$

Since θ is the belief factor for the predicted measurement, Equation (24) indicates that when NPD error is high with a large $R_{t}$, ${\hat{z}}_{t}$ offers more contribution than the noisy measurement. In other words, when the prediction covariance $Q_{t}$ is larger than $R_{t}$, our method should assign more belief to $z_{t}$. In this case, ${\hat{z}}_{t}$ is useless and can introduce more estimation error. Thus, when the measurement error is quite small and the prediction error exceeds the measurement error, using our method will introduce extra estimation error. Then, the conventional methods can outperform our algorithms. This means that our method is useful when the measurement uncertainty is higher than the predicted state uncertainty, and the optimal θ exists. Fortunately, in the indoor wireless tracking system, the measurement uncertainty and outlier values are always high. In addition, if the measurement noise is low, the prediction error is also small, due to the performance of the Bayesian filtering estimation. Therefore, our method can effectively improve the estimation accuracy in the noisy environment.

## 5. Adaptive Particle Filter

According to the architecture in Figure 3, we integrate our adaptation method with three main PFs, which are BPF, GPF and CPF, and design new PF versions: adaptive BPF (A-BPF), adaptive GPF (A-GPF) and adaptive CPF (A-CPF).

### 5.1. Adaptive Bootstrap Particle Filter

In A-BPF, the prediction state ${\hat{x}}_{t}$ is obtained through:

(25) $${\hat{x}}_{t}=f_{t}\left(x_{t-1}\right)$$

where the previous state $x_{t-1}$ is calculated based on: $x_{t-1}=\sum_{i=1}^{N_{s}}w_{t-1}^{i}x_{t-1}^{i}$. Then, we obtain the predicted measurement ${\hat{z}}_{t}$ according to Equation (13).

When the measurement $z_{t}$ is available, the adapted measurement likelihood $p(z_{t}|x_{t}^{i})$ for each particle is calculated as Equation (14). Additionally, then, the particle weight is calculated and normalized as $w_{t}^{i}\propto w_{t-1}^{i}p_{AL}\left(z_{t}|x_{t}^{i}\right)$, which determines the posterior PDF of the estimated state $x_{t}$. Finally, $x_{t}$ is attained: $x_{t}=\sum_{i=1}^{N_{s}}w_{t}^{i}x_{t}^{i}$. The importance sampling and resampling parts are still the same, and the predicted measurement and optimal θ is easy to obtain based on the analytical formulation. Therefore, our likelihood adaptation does not introduce much computational complexity to the original PFs. The algorithm of A-BPF is presented in Algorithm 1. Our adaptation method is implemented in the importance sampling part and the weight adaptation part of PF.

### 5.2. Adaptive Gaussian Particle Filter

The GPF approximates the estimated PDF by Gaussian distributions using the PF method. The GPF assumes that PDF of the state follows a Gaussian distribution, and it samples particles according to the estimated PDF [11]. Therefore, only the mean and covariance of the estimated PDF are calculated and propagated. Due to the simplicity, the GPF is widely used in distributed PF applications [12].

Particles are drawn from the Gaussian distribution functions, ${\left\{x_{t}^{i}\right\}}_{i=1}^{N_{s}}∼N\left(\mu_{t},Q_{t}\right)$, where $\mu_{t}$ is the mean value of estimated state and $Q_{t}$ is the covariance of PDF. The Gaussian PDF evolves according to the transition model: $x_{t}=f_{t}\left(x_{t-1}\right)$. Thus, the covariance is assumed to propagate to the next time step, which is $Q_{t|t-1}=Q_{t-1}$. Then, the initial weight for each particle is calculated as:

(26) $$w_{t}^{i}\propto N\left(x_{t}=x_{t}^{i},{\hat{x}}_{t},Q_{t|t-1}\right)$$

When the measurements are available, the weight for each particle updates:

(27) $$w_{t}^{i}\propto p\left(z_{t}|x_{t}^{i}\right)N\left(x_{t}=x_{t}^{i},{\hat{x}}_{t},Q_{t|t-1}\right)$$

where $p(z_{t}|x_{t}^{i})$ is the likelihood.

**Algorithm 1** Adaptive bootstrap particle filter (A-BPF).Prediction: ${\hat{x}}_{t}=f_{t}\left(x_{t-1}\right)$;Prior Measurement: ${\hat{z}}_{t}=h_{t}\left({\hat{x}}_{t}\right)$;//Importance SamplingDraw: ${\left\{x_{t}^{i}∼p\left(x_{t}^{i}|x_{t-1}^{i}\right)\right\}}_{i=1}^{N_{s}}$;//Measurement Adaptation**for** particle $i=1:N_{s}$ **do**    Likelihood: $p_{AL}\left(z_{t}|x_{t}^{i}\right)=p\left(\theta ⊙{\hat{z}}_{t}+\left(1-\theta \right)⊙z_{t}-h_{t}\left(x_{t}^{i}\right)\right)$;    Weight: $w_{t}^{i}=w_{t-1}^{i}p_{AL}\left(z_{t}|x_{t}^{i}\right)$;**end for**Normalizing: $w_{t}^{i}=\frac{w_{t}^{i}}{\sum_{i=1}^{N_{s}}w_{t}^{i}}$;Resampling: ${\left\{x_{t}^{i},w_{t}^{i}\right\}}_{i=1}^{N_{s}}$;State Estimation ${\bar{x}}_{t}=\sum_{i=1}^{N_{s}}w_{t}^{i}x_{t}^{i}$;

In the A-GPF, the LF fuses both the prior information and current data based on the DGM. The optimal θ is derived based on the previous estimated $Q_{t|t-1}$ and current NPD covariance $R_{t}$. Thus, calculating the optimal θ is a recursive procedure in A-GPF. The procedure of A-GPF is illustrated in Algorithm 2.

**Algorithm 2** Adaptive Gaussian particle filter (A-GPF).Prediction: ${\hat{x}}_{t}=f_{t}\left(x_{t-1}\right)$Calculate ${\hat{z}}_{t}$ based on Equation (13)Randomly Draw: ${\left\{x_{t}^{i}\right\}}_{i=1}^{N_{s}}∼N\left(\mu_{t},Q_{t|t-1}\right)$$\theta =\frac{R_{t}}{\frac{\partial h_{t}\left(x_{t}\right)}{\partial x_{t}}Q_{t|t-1}{\left[\frac{\partial h_{t}\left(x_{t}\right)}{\partial x_{t}}\right]}^{T}+R_{t}}$**for** particle $i=1:N_{s}$ **do**    Likelihood: $p_{AL}\left(z_{t}|x_{t}^{i}\right)=p\left(\theta ⊙{\hat{z}}_{t}+\left(1-\theta \right)⊙z_{t}-h_{t}\left(x_{t}^{i}\right)\right)$;    Weight: $w_{t}^{i}=w_{t-1}^{i}p_{AL}\left(z_{t}|x_{t}^{i}\right)$;    Gaussian Distribution Estimation:    Mean: ${\bar{x}}_{t}={\bar{\mu }}_{t}=\sum_{i=1}^{N_{s}}w_{t}^{i}x_{t}^{i}$    Covariance: ${\bar{Q}}_{t}=\sum_{i=1}^{N_{s}}w_{t}^{i}\left({\bar{\mu }}_{t}-x_{t}^{i}\right){\left({\bar{\mu }}_{t}-x_{t}^{i}\right)}^{T}$**end for**

The difference between A-BPF and A-GPF is: the prediction error in A-BPF follows an arbitrary assumed distribution, whereas A-GPF uses the Gaussian distribution to indicate such a distribution. The assumed distribution in A-BPF is obtained based on the statistical analysis, and the estimation of A-BPF can be accurate if the assumed distribution is correct.

### 5.3. Adaptive Constraint Particle Filter

The CPF randomly samples particles not only based on both the assumed distribution and some constraint conditions, e.g., $x_{t}^{i}\in c\left(x_{t},z_{t}\right)$, where $c(x_{t},z_{t})$ is the constraint functions [9]. The constraint conditions guarantee the particle generated in the target region with a very high probability by allocating a higher previous weight. In this case, both the previous weight and adaptive parameters are maintained during the estimation, where the complexity is still high. Thus, we use the constraint conditions only to sample the particles, and the weights are calculated according to the adaptive likelihoods. Then, the complexity of A-CPF is reduced.

The constraint conditions can be set up according to different applications. We will detail the conditions for the range-based target tracking in the next section. After sampling the particles, the prediction is obtained based on the prediction function. Then, our adaptation method is used, and the weight calculation follows the same procedure as A-BPF, which is illustrated in Algorithm 3. The major difference between A-BPF and A-CPF is the additional constrains for particles, which can improve the performance of BPF with additional information.

**Algorithm 3** Adaptive constraint particle filter (A-CPF).Constraint Sampling: ${\left\{x_{t}^{i}\in c\left(x_{t},z_{t}\right)\right\}}_{i=1}^{N_{s}}$Prediction: ${\hat{x}}_{t}=f_{t}\left(x_{t-1}\right)$Calculate ${\hat{z}}_{t}$ based on Equation (13)**for** particle $i=1:N_{s}$ **do**    Likelihood: $p_{AL}\left(z_{t}|x_{t}^{i}\right)=p\left(\theta ⊙{\hat{z}}_{t}+\left(1-\theta \right)⊙z_{t}-h_{t}\left(x_{t}^{i}\right)\right)$;    Weight: $w_{t}^{i}=w_{t-1}^{i}p_{AL}\left(z_{t}|x_{t}^{i}\right)$;    Normalizing: $w_{t}^{i}=\frac{w_{t}^{i}}{\sum_{i=1}^{N_{s}}w_{t}^{i}}$;    Resampling: ${\left\{x_{t}^{i},w_{t}^{i}\right\}}_{i=1}^{N_{s}}$;**end for**

### 5.4. Performance Comparison

Note that the performances of the proposed APFs are different and suitable for different scenarios. In general, all of the APFs can effectively reduce the estimation error in the high noise environment, which will be proven in Section 6. The A-BPF reduces the estimation error by using arbitrary prior information. Such information is set up since the initial sampling scheme. If the sampling scheme and prior information are accurate, the estimation results can be good. However, the system cannot adjust the prior information at every time step. Thus, the A-BPF cannot further reduce the error if the measurement error is small. The A-GPF is quite suitable for adjusting itself during the recursive estimation. At each time step, A-GPF calculates the estimation covariance using the current particles and propagates to the next time step as the prior information. This is quite flexible for a dynamic environment. However, the disadvantage of the A-GPF is that there is some error in the covariance calculation. Thus, if the measurement error is too high, the performance of the A-GPF is not as good as A-BPF. The most robust feature for the dynamic environment is A-CPF, which employs constraint conditions to restrict error. In a high noise environment, the estimation error of the A-CPF can be controlled. However, the A-CPF has an inherent error from the constraint condition. Thus, it is still not as good as the A-BPF if the measurement error is small.

## 6. CRLB Analysis

In this section, we provide the benefits of the adaptive PFs by deriving the CRLB. The CRLB, which is given by the inverse of the Fisher information matrix (FIM), sets the lower limit for the variance (or covariance matrix) of any unbiased estimators of an unknown parameter (or unknown parameters) [35].

### 6.1. FIM of the Adapted Likelihood

For the adaptive PFs, the prior information is integrated into the likelihood function. If the measurement noise also follows a Gaussian distribution, we can easily derive the covariance of adapted measurement:

(28) $$\begin{array}{cc} \mathrm{Cov}({\bar{z}}_{t}) & =E\{\left(h_{t}\left(x_{t}\right)-{\bar{z}}_{t}\right){\left(h_{t}\left(x_{t}\right)-{\bar{z}}_{t}\right)}^{T}\}\\ & =E\{||\theta \frac{\partial h_{t}\left(x_{t}\right)}{\partial x_{t}}q_{t}+\left(1-\theta \right)n_{t}+w_{t}|{|}^{2}\}\\ & =\theta \frac{\partial h_{t}\left(x_{t}\right)}{\partial x_{t}}Q_{t}{\left[\frac{\partial h_{t}\left(x_{t}\right)}{\partial x_{t}}\right]}^{T}\theta^{T}+\left(1-\theta \right)R_{t}{\left(1-\theta \right)}^{T}+W_{t}\\ & ={\bar{R}}_{t} \end{array}$$

If the measurement noises are independent of each anchor, the covariance matrix can be simplified as ${\bar{R}}_{t}=diag_{N\times N}\left({\bar{R}}_{j}\right)$, where ${\bar{R}}_{j}$ can be derived independently in each anchor. Then, $I_{D}\left(x_{t}\right)$ is formulated as:

(29) $$\begin{array}{c} \begin{array}{c} I_{D}\left(x_{t}\right)=\left(\begin{array}{cc} \sum_{j=1}^{N}{\bar{R}}_{j}^{-1}{\left(\frac{\partial h_{t}^{j}\left(x_{t}\right)}{\partial p_{x}^{t}}\right)}^{2} & \sum_{j=1}^{N}{\bar{R}}_{j}^{-1}\frac{\partial h_{t}^{j}\left(x_{t}\right)}{\partial p_{x}^{t}}\frac{\partial h_{t}^{j}\left(x_{t}\right)}{\partial p_{y}^{t}}\\ \sum_{j=1}^{N}{\bar{R}}_{j}^{-1}\frac{\partial h_{t}^{j}\left(x_{t}\right)}{\partial p_{x}^{t}}\frac{\partial h_{t}^{j}\left(x_{t}\right)}{\partial p_{y}^{t}} & \sum_{j=1}^{N}{\bar{R}}_{j}^{-1}{\left(\frac{\partial h_{t}^{j}\left(x_{t}\right)}{\partial p_{y}^{t}}\right)}^{2} \end{array}\right) \end{array} \end{array}$$

for the detailed formulation, please refer to the Appendix.

### 6.2. FIM of Recursive Estimation

The FIM of recursive Bayesian estimation has been initially formulated by Petr *et al.* [36], which is given as:

(30) $$I\left(x_{t}\right)=A-B{\left(I\left(x_{t-1}\right)+C\right)}^{-1}B^{T}$$

where

(31) $$\begin{array}{cc} A & =I_{P}\left(x_{t}\right)+I_{D}\left(x_{t}\right)=\frac{\partial f_{t}\left(x_{t}\right)}{\partial x_{t}}Q_{t}^{-1}{\frac{\partial f_{t}\left(x_{t}\right)}{\partial x_{t}}}^{T}+I_{D}\left(x_{t}\right)\\ B & =-\frac{\partial f_{t}\left(x_{t}\right)}{\partial x_{t}}Q_{t}^{-1}\\ C & =\frac{\partial f_{t}\left(x_{t}\right)}{\partial x_{t}}Q_{t}^{-1}{\frac{\partial f_{t}\left(x_{t}\right)}{\partial x_{t}}}^{T} \end{array}$$

where $I(x_{t})$ is a part of $I({\left[x_{t}\;x_{t-1}\right]}^{T})$; A, B and C are parts of $I({\left[x_{t}\;x_{t-1}\right]}^{T})$, which represent the previous and current information. For the detailed description, please refer to the Appendix.

### 6.3. Analytical Results

**Figure 4:**
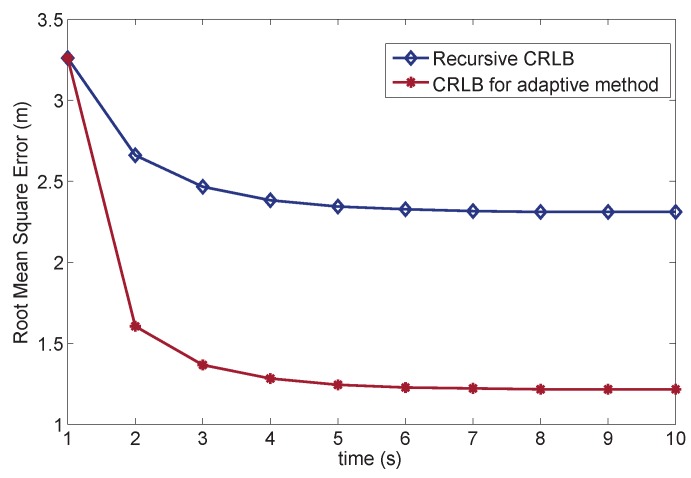
Cramér–Rao lower bound for target tracking.

We set up several simulations to evaluate the performance improvement of the adaptive PF. The playing field is 100×100 m${}^{2}$. We randomly deploy 100 sensors. The target chooses a random path walking through the playing field. The range data are obtained through the TOA technique, whose range information is formulated according to Equation (4). The EPD for each sensor follows zero mean Gaussian distribution $w_{t}^{j}∼\sigma_{w}^{2}$, where $\sigma_{w}=1$ m. Additionally, set the NPD of each sensor as $n_{t}^{j}∼N\left(1,\sigma_{n}^{2}\right)$, where $\sigma_{n}=3$ m. Root mean square error (RMSE) is mainly compared in the simulation as the estimation accuracy metric. For the CRLB analysis, the RMSE indicates the root value of the unbiased covariance. To compare with our proposed method, we use the common recursive CRLB form in [36], where the measurement noise vector is the combination of the EPD and NPD $v_{t}∼N\left(1,R_{t}+W_{t}\right)$. It should be noted that the the measurement noise in the simulation is not simply the sum of two distribution. EPD is the general error distribution, which is added during the whole simulation. However, NPD is applied to represent outlier values of the simulation. Thus, during each time step, we randomly add an NPD error or not. In this case, the NPD error is a few outlier values that can influence the measurement, and DGM must be applied for the PF estimation. The sequential estimation results are illustrated in Figure 4.

**Figure 5:**
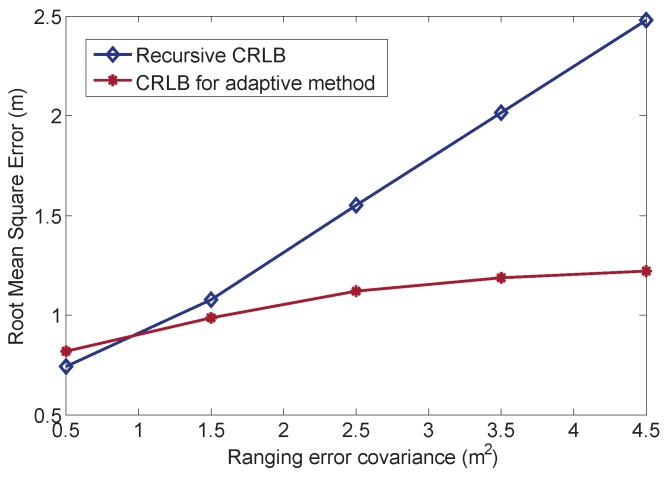
RMSE results of the Cramér–Rao lower bound with a multiple NPD environment.

We also adapt the measurement noise environment to examine the performance change for each process. The NPD covariance for each anchor changes from $R_{j}={\left(0.5\;m\right)}^{2}$ to $R_{j}={\left(5.5\;m\right)}^{2}$. The root mean squared error (RMSE) results of the two CRLBs are depicted in Figure 5. When the NPD covariance is small, the adaptive method cannot improve estimation, but increases the estimation error. In this case, it would rather use only the measurement data to obtain the estimation. However, with increasing the NPD covariance, the adaptive method improves the estimation gradually and outperforms the recursive Bayesian method when the error is quite high. Thus, our method is feasible for a harsh environment with high uncertainty.

## 7. Range-Based Target Tracking Simulation and Experiment

### 7.1. Simulation Setup

**Figure 6:**
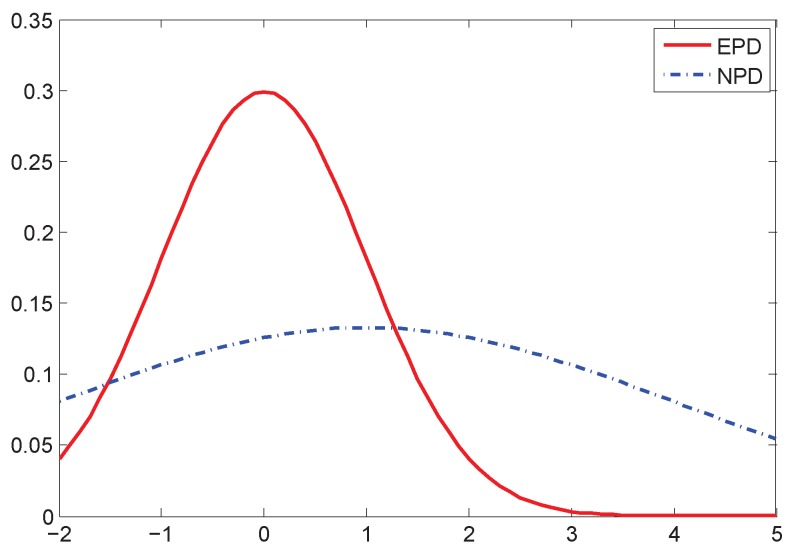
The dynamic Gaussian model (DGM) pattern in the the simulation.

Our scheme is evaluated in the simulation of target tracking using the wireless sensor networks. We randomly deploy 100 sensors in a two-dimensional square 100×100 m${}^{2}$ region. One target runs through a random path with a constant speed. The target periodically broadcasts the request signals to the sensors, and the sensors respond to the request. Then, the ranging values are calculated according to the time-of-arrival (TOA) of the responses. Each sensor node *j* is assigned coordinations ${\left[s_{x}^{j},s_{y}^{j}\right]}^{T}$, and the target state at time *t* is $x_{t}={\left[p_{x}^{t},p_{y}^{t},v_{x}^{t},v_{y}^{t}\right]}^{T}$, where $\left[p_{x}^{t},p_{y}^{t}\right]$ is the position vector, $\left[v_{x}^{t},v_{y}^{t}\right]$ is the velocity vector and *T* is the transpose operator. In the simulation, we assume that the measurement noise consists of the EPD and the NPD values, and the measurement noise for each sensor node is identical independent distributed (i.i.d). The EPD noise follows the zero mean Gaussian distribution, where $w_{t}^{j}∼N\left(0,\sigma_{w}^{2}\right)$ and $\sigma_{w}=1$ m. Additionally, the NPD noise also follows a Gaussian distribution, where $n_{t}^{j}∼N\left(1,\sigma_{n}^{2}\right)$ and $\sigma_{n}=3$ m. Then, the DGM can be illustrated as shown in Figure 6.

### 7.2. Particle Filter Modeling

We employ a linear model as the prediction function, and Equation (1) can be expressed as:

(32) $$x_{t}=F_{t}x_{t-1}+q_{t}$$

where $F_{t}$ is the linear transition matrix:

(33) $$F_{t}=\left(\begin{array}{cccc} 1 & 0 & \Delta T & 0\\ 0 & 1 & 0 & \Delta T\\ 0 & 0 & 1 & 0\\ 0 & 0 & 0 & 1 \end{array}\right)$$

where ΔT indicates the time interval.

In the particle sampling stage, the samples are initially randomly generated and propagated according to the linear transition model in BPF and A-BPF. In GPF and A-GPF, the particles are sampled based on the updated PDF. In CPF, the particles are constrained to some conditions. For range-based localization systems, the constraint region can be drawn by the bounding box algorithm, which is demonstrated to be robust to measurement noise [37]. Using $z_{t}^{j}$ to denote the *j*-th range measurement for node *j* with the position ${\left[s_{x}^{j},s_{y}^{j}\right]}^{T}$ in the two-dimensional playing field, then the geometric constraint region is: $max{\left\{s_{x}^{j}-z_{t}^{j}\right\}}_{j=1}^{N}\leq p_{x}^{t}\leq min{\left\{s_{x}^{j}+z_{t}^{j}\right\}}_{j=1}^{N}$ and $max{\left\{s_{y}^{j}-z_{t}^{j}\right\}}_{j=1}^{N}\leq p_{y}^{t}\leq min{\left\{s_{y}^{j}+z_{t}^{j}\right\}}_{j=1}^{N}$, where *N* is the number of sensor nodes. Then, the particles are sampled within this region.

### 7.3. Optimal Belief Factor for A-BPF

**Figure 7:**
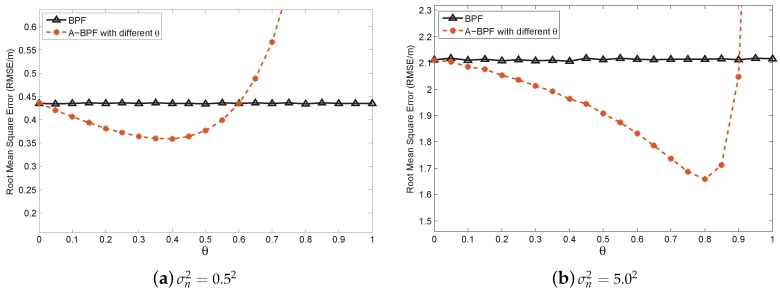
Estimation error comparison for different algorithms with different measurement error variances.

First, we compare the estimation accuracy of BPF to our algorithm, A-BPF, to analyze the relationship between θ and the estimation accuracy. We implement A-BPF and vary θ from 0 to 1 to verify whether θ has an optimal value with minimum estimation error. The algorithms are tested in the different measurement noise scenarios, in which the NPD noise variance $\sigma_{n}^{2}$ is tuned from $0.5^{2}$ to $5^{2}$. The results of our simulation are averaged by over 1000 Monte-Carlo trials. We randomly deploy sensor nodes in every trial. In this simulation, all algorithms generate 1000 particles at each time step *t*. Figure 7 illustrates the root mean square error (RMSE) of each algorithm with some different measurement noise scenarios.

In Figure 7, the solid line represents the RMSE of BPF, and the dashed line indicates the RMSE of A-BPF with different θ, in which θ changes from 0 to 1 with an interval of 0.05. When θ=0, A-BPF totally believes the noisy measurement and has the same accuracy as BPF. The RMSE of A-BPF is decreasing when θ increases from 0, which indicates that our adaptation method can improve the measurement likelihood and estimation accuracy. As indicated in each figure in Figure 7, A-BPF has a minimum RMSE with optimal θ in different measurement noise scenarios. When θ is larger than the optimal value, the RMSE of A-BPF begins to increase. Additionally, when θ approaches 1, it is over tuned and causes high estimation error.

When the measurement nose is low, e.g., in Figure 7a, A-BPF does not improve much, even with the optimal θ. However, when the measurement noise is quite large, e.g., in Figure 7b, A-BPF can effectively reduce the RMSE with the optimal θ. Additionally, the gap between A-BPF and BPF is much larger in Figure 7b than in Figure 7a. Besides, the optimal θ increases when the NPD noise rises, e.g., in Figure 7a, θ is 0.3, and it becomes 0.8 in Figure 7b.

**Figure 8:**
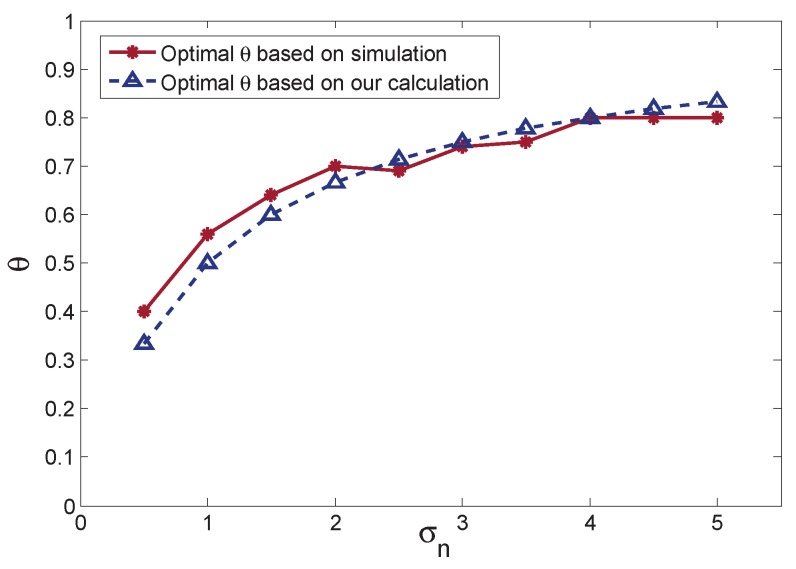
Optimal θ comparison between the simulation results and our calculation based on Equation (24).

The comparison of θ obtained in the simulation and derived in A-BPF is depicted in Figure 8. The solid curve is the optimal θ in the simulations, and the dashed curve is the optimal θ based on Equation (24) with different measurement noise. As shown in Figure 8, the optimal θ based on our calculation in Equation (24) is slightly different from the simulation results, because the optimal θ is derived according to the approximation in Equation (20). Since the optimal θ is approaching the simulation results, it is still suitable for implementation. Figure 8 also depicts that the optimal θ increases with the rise of measurement noise. It testifies for Equation (24) that θ rises when the measurement noise becomes larger. In this case, A-BPF assigns more belief for the predicted measurement for adaptation.

### 7.4. Root Mean Square Error Comparison

**Figure 9:**
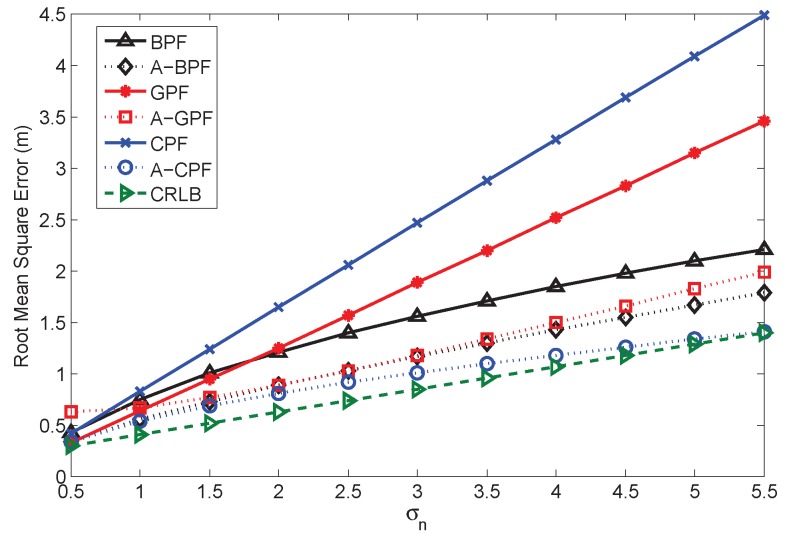
Comparing the performances of different algorithms.

Figure 9 illustrates the RMSE comparison for BPF, A-BPF, GPF, A-GPF, CPF and A-CPF, and the CRLB for the adaptive filters is also depicted as the optimal indicator. The measurement noise covariance varies from $0.5^{2}$ to $5.5^{2}$, and we use the optimal θ based on Equation (24) as the belief factor in the adaptive PFs. When the measurement error is small, the performances of the PFs are similar. Furthermore, we can find that the adaptive PFs have slightly higher error than the original PFs when the noise is too low. This is due to the inherent sampling method, which generate samples with higher statistical covariance than the measurement noise. In this case, our adaptation method relies on such covariance and causes extra error in the final estimation, which is as illustrated in the previous sections. When the error increases, the estimation errors of BPF, GPF and CPF rise accordingly. Especially, CPF has the highest RMSE due to the imprecise likelihood calculation, although it has constraints. This indicates that the measurement noise is the major impact that influences the estimation performance of PFs. The adaptive PFs have better performance. A-BPF and A-GPF have similar estimation accuracy. The RMSE of A-CPF is the lowest and mostly approaching the CRLB.

**Figure 10:**
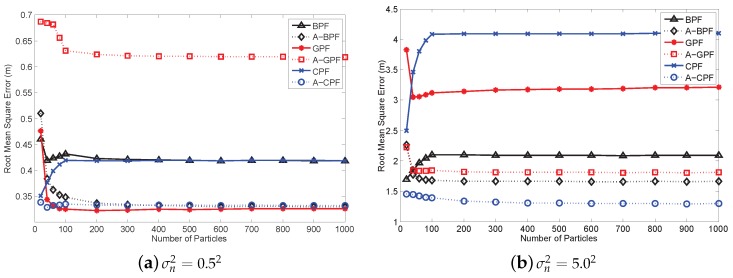
RMSE comparison for different algorithms with different numbers of particles.

We also compare the estimation performance of PFs with varying the particle numbers. As Crisan *et al.* indicate, the estimation error should converge to zero with the increasing number of particles [38]. However, simulation results show that the estimation accuracy is corrupt with high measurement noise. When the error is small, as illustrated in Figure 10a, the RMSEs of PFs can converge to a very low value with the increased particle number, except BPF and CPF, which illustrates that the measurement noise can influence the convergence of PFs, although not much. In this case, our adaptive method does not improve much, and A-GPF even has a higher RMSE than GPF. When the measurement noise begins to rise, increasing the particle number of the original PFs cannot improve the estimation accuracy. In Figure 10b, the RMSEs of BPF and CPF begin to rise when the number of particles exceed 100, and GPF does not improve either. This implies that high measurement error leads to a significantly inaccurate likelihood calculation, which degrades the estimation. In addition, CPF outperforming BPF, which is mentioned in [9], only occurs in the low noise case. However, our adaptive method can improve the estimation accuracy and make PFs converge to a low RMSE. In Figure 10, RMSEs of three adaptive PFs decrease with the rising particle number. RMSEs can converge to a very low value even when the measurement error is high. Therefore, our method can reduce the imprecise measurement effect and achieve a better performance.

### 7.5. The Real Indoor Experiment Test-Bed

**Figure 11:**
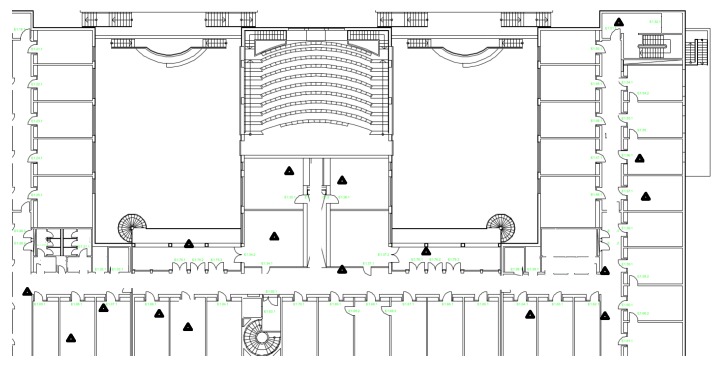
Building layout for indoor localization experiment and the robot trajectory. The triangles mark the positions of sensor nodes, which are placed either in the offices or along the corridor.

We also employ a reference system for indoor localization test-beds to examine our proposed algorithms. In this system, we deployed 17 wireless sensor nodes, either along the corridor or in the offices of the research building. A robot carrying a sensor node as a target moved along the corridor of the building with constant speed while recording its own positions [30]. In the previous simulations, we assume that the noise follows an independent identical distribution. However, in the real indoor environment, the wireless signals are propagated through complicated channels. The LOS and NLOS range measurements are mixed for a single target. Thus, a specific distribution for each sensor or anchor is necessary to derive accurate estimation. We still use the DGM to model the measurement noise. All sensors are integrated with the nanoPAN 5375 RF module with a 2.4-GHz transceiver and a 1-Mbps data rate for range measurement, LPC 2387 as the micro-controller and a CC1101 900-MHz transceiver as the radio transceiver for communication. The data collected from sensor nodes are also range measurement values, which are based on TOA. There is an inherent error when the 3D localization is projected into the 2D map, due to the height difference between the sensors and target [39]. We adapt the heights of the sensors to be the the same as the robot to reduce the impact of the height difference in the 3D world. In this case, we can draw an exact 2D trajectory within the map. Figure 11 depicts the map of our experimental building. The triangles, which are randomly deployed, mark the sensor node positions.

**Figure 12:**
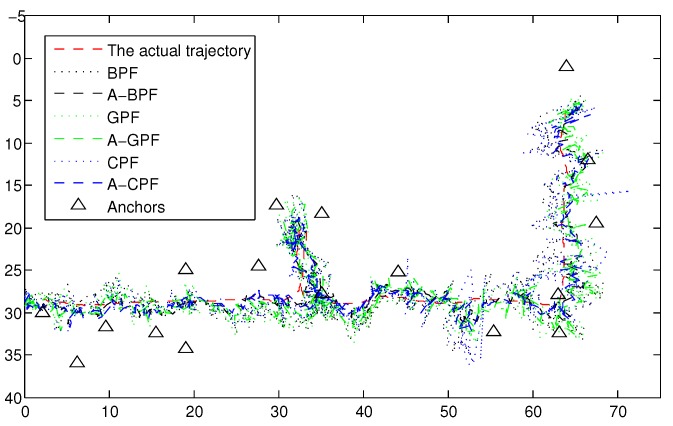
Trajectories for the indoor target tracking.

**Figure 13:**
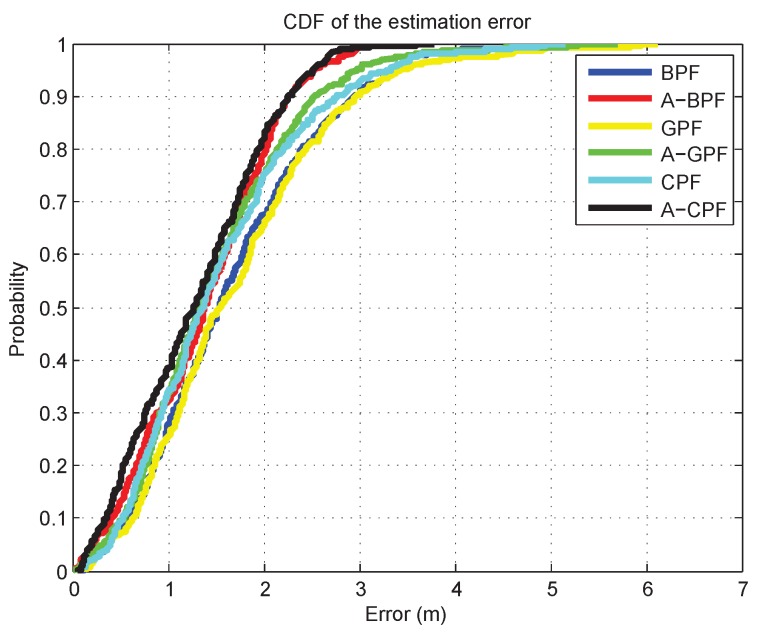
Overall cumulative distribution functions of particle filters (PFs) for both trajectories.

We also implement the six algorithms mentioned above to compare their performance. Each algorithm maintains 200 particles, and several trajectories are examined. In Figure 12, we present one estimated trajectory. We compare the estimation performance by evaluating the RMSE. The RMSE results of the six algorithms are listed in Table 1. As shown in Table 1, the mean average errors (MAE), which are the average performance, for adaptive PF are less than the original PFs. Additionally, the adaptive PFs have about 1 m less than the original PFs in the RMSE. The max error, which is the worst case for estimation, in the original PFs is more than 6 m and even 8 m in CPF. However, adaptive PFs have about less than 6.91 m, and the max error can even reach 4.0649 m in A-BPF in Table 1, which is much more precise than the original PFs. The overall cumulative distribution functions (CDF) for both trajectories are plotted in Figure 13. Based on the comparison, our adaptive PFs generally outperform the original PFs and show robust performance in the dynamic indoor environment.

## 8. Conclusions

In this paper, we use a DGM, which combines the EPD and NPD, to describe the hybrid LOS/NLOS ranging noise in the indoor environment. Based on this model, we propose an adaptation method by introducing the predicted measurement and its belief factor θ. The optimal θ is derived and implemented into our proposed adaptive algorithms, A-BPF, A-GPF and A-CPF. The estimation performance of the adaptive PFs is proven by the CRLB analysis. In the simulation, we observed that some analytical conclusion for PFs are not suitable for the high measurement noise scenarios, and we verify that the adaptive PFs improve the estimation accuracy with different measurement noise environments and that the optimal θ derived in our method approaches the actual value. The real indoor localization experimental results indicate that, comparing to the original PFs, our algorithms can effectively reduce the estimation error, especially in a high measurement noise environment. Besides, A-CPF is more accurate for target tracking than the other filters.

## Figures and Tables

**Table 1:** Trajectory I: Performance comparison. BPF, bootstrap particle filter; A-BPF, adaptive BPF; GPF, Gaussian particle filter; CPF, constraint particle filter.

Algorithm	MAE (m)	RMSE (m)	Min Error (m)	Max Error (m)
BPF	0.4375	2.3637	0.0649	6.9100
A-BPF	0.2092	1.2907	0.0340	4.0649
GPF	0.4078	2.1231	0.0723	6.2308
A-GPF	0.1612	1.6453	0.0443	5.0614
CPF	0.2455	2.0439	0.0397	8.4929
A-CPF	0.1517	1.3395	0.0506	4.6814

## Appendix A. CRLB Derivation

The CRLB, which is given by the inverse of the Fisher information matrix (FIM), sets the lower limit for the variance (or covariance matrix) of any unbiased estimators of an unknown parameter (or unknown parameters) [35].

If $p(x_{t},z_{t})$ denotes the joint PDF of observations $z_{t}$ and the state $x_{t}$, then the score function is defined as the gradient of its log-likelihood:

(A1) $$U\left(x_{t}\right)=\nabla \ln p\left(x_{t},z_{t}\right)=\frac{\partial \ln p(x_{t},z_{t})}{\partial x_{t}}$$

The FIM, $I(x_{t})$, is the variance of the score function:

(A2) $$I\left(x_{t}\right)=E\left\{{\left[\frac{\partial \ln p(x_{t},z_{t})}{\partial x_{t}}\right]}^{2}\right\}$$

and CRLB is just the inverse of FIM; the estimation covariance cannot be lower than it:

(A3) $${\mathrm{Cov}}_{x_{t}}\left(\tilde{x_{t}}\right)⪰{\left\{I\left(x_{t}\right)\right\}}^{-1}$$

Note that the CRLB relies on the system model and the noise distribution. In general, using different information can lead to different results. For instance, if the system only applies the measurement model, the CRLB is higher than the recursive CRLB, which applies the previous information iteratively. In our work, we combine the previous information into the measurement model. When the model and related error distribution are changed, a new CRLB is derived accordingly.

Since $p\left(x_{t},z_{t}\right)=p\left(z_{t}|x_{t}\right)p\left(x_{t}\right)$ based on the Bayesian theorem, it is easily seen that $I(x_{t})$ can be decomposed into two parts:

(A4) $$I\left(x_{t}\right)=I_{D}\left(x_{t}\right)+I_{P}\left(x_{t}\right)$$

where $I_{D}\left(x_{t}\right)$ represents the information obtained from measurement data and $I_{P}\left(x_{t}\right)$ represents the prior information.

Firstly, we decompose $I_{D}\left(x_{t}\right)$ using the chain rule as:

(A5) $$I_{D}\left(x_{t}\right)=H\cdot I_{H}\cdot H^{T}$$

where $H=[\nabla_{x_{t}}h_{t}\left(x_{t}\right)]$ is the Jacobian matrix of $x_{t}$:

(A6) $$H=\left(\begin{array}{ccc} \frac{\partial h_{t}^{1}\left(x_{t}\right)}{\partial p_{x}^{t}} & \ldots & \frac{\partial h_{t}^{N}\left(x_{t}\right)}{\partial p_{x}^{t}}\\ \frac{\partial h_{t}^{1}\left(x_{t}\right)}{\partial p_{y}^{t}} & \ldots & \frac{\partial h_{t}^{N}\left(x_{t}\right)}{\partial p_{y}^{t}} \end{array}\right)$$

where *N* indicates the dimension of vector $z_{t}$, $x_{t}=\left[p_{x}^{t}\;p_{y}^{t}\right]$. Note that we employ the measurement function according to Equation (4), then we have:

(A7) $$\left\{\begin{array}{c} \frac{\partial h_{t}^{j}\left(x_{t}\right)}{\partial p_{x}^{t}}=\frac{p_{x}^{t}-a_{x}^{j}}{\sqrt{{\left(p_{x}^{t}-a_{x}^{j}\right)}^{2}+{\left(p_{y}^{t}-a_{y}^{j}\right)}^{2}}}\\ \frac{\partial h_{t}^{j}\left(x_{t}\right)}{\partial p_{y}^{t}}=\frac{p_{y}^{t}-a_{y}^{j}}{\sqrt{{\left(p_{x}^{t}-a_{x}^{j}\right)}^{2}+{\left(p_{y}^{t}-a_{y}^{j}\right)}^{2}}} \end{array}\right.$$

and $I_{H}$ is the FIM conditioned on $h_{t}\left(x_{t}\right)$:

(A8) $$I_{H}=E\left\{\nabla_{h_{t}}\ln p\left(z_{t}|x_{t}\right){\left[\nabla_{h_{t}}\ln p\left(z_{t}|x_{t}\right)\right]}^{T}\right\}$$

which is the measurement covariance $R_{t}^{-1}$:

(A9) $$I_{H}=R_{t}^{-1}=\left(\begin{array}{ccc} R_{t}^{1} & & \\ & \ddots & \\ & & R_{t}^{N} \end{array}\right)$$

then the $I_{D}\left(x_{t}\right)$ for conventional CRLB is:

(A10) $$I_{D}\left(x_{t}\right)=\left(\begin{array}{cc} \sum_{j=1}^{N}R_{j}^{-1}{\left(\frac{\partial h_{t}^{j}\left(x_{t}\right)}{\partial p_{x}^{t}}\right)}^{2} & \sum_{j=1}^{N}R_{j}^{-1}\frac{\partial h_{t}^{j}\left(x_{t}\right)}{\partial p_{x}^{t}}\frac{\partial h_{t}^{j}\left(x_{t}\right)}{\partial p_{y}^{t}}\\ \sum_{j=1}^{N}R_{j}^{-1}\frac{\partial h_{t}^{j}\left(x_{t}\right)}{\partial p_{x}^{t}}\frac{\partial h_{t}^{j}\left(x_{t}\right)}{\partial p_{y}^{t}} & \sum_{j=1}^{N}R_{j}^{-1}{\left(\frac{\partial h_{t}^{j}\left(x_{t}\right)}{\partial p_{y}^{t}}\right)}^{2} \end{array}\right)$$

then, we derive the FIM of the adaptive likelihood and recursive formulation based on Equation (A10).

### Appendix A.1. FIM of the Adapted Likelihood

For the adaptive PFs, the prior information is integrated into the likelihood function. Since the measurement is adapted by the prior information, $I_{D}$ is changed by using the adapted measurement ${\bar{z}}_{t}$, which is the combination of predicted measurement and observed data. Then, the error covariance does not follow the original error distribution, but changes accordingly. If the measurement noise also follows a Gaussian distribution, we can easily derive the covariance of adapted measurement:

(A11) $$\begin{array}{cc} \mathrm{Cov}({\bar{z}}_{t}) & =E\{\left(h_{t}\left(x_{t}\right)-{\bar{z}}_{t}\right){\left(h_{t}\left(x_{t}\right)-{\bar{z}}_{t}\right)}^{T}\}\\ & =E\{||\theta \frac{\partial h_{t}\left(x_{t}\right)}{\partial x_{t}}q_{t}+\left(1-\theta \right)n_{t}+w_{t}|{|}^{2}\}\\ & =\theta \frac{\partial h_{t}\left(x_{t}\right)}{\partial x_{t}}Q_{t}{\left[\frac{\partial h_{t}\left(x_{t}\right)}{\partial x_{t}}\right]}^{T}\theta^{T}+\left(1-\theta \right)R_{t}{\left(1-\theta \right)}^{T}+W_{t}\\ & ={\bar{R}}_{t} \end{array}$$

If the measurement noise is independent of each anchor, the covariance matrix can be simplified as ${\bar{R}}_{t}=diag_{N\times N}\left({\bar{R}}_{j}\right)$, where ${\bar{R}}_{j}$ can be derived independently in each anchor. Similar to Equation (A10), $I_{D}\left(x_{t}\right)$ is formulated as:

(A12) $$\begin{array}{c} I_{D}\left(x_{t}\right)=\left(\begin{array}{cc} \sum_{j=1}^{N}{\bar{R}}_{j}^{-1}{\left(\frac{\partial h_{t}^{j}\left(x_{t}\right)}{\partial p_{x}^{t}}\right)}^{2} & \sum_{j=1}^{N}{\bar{R}}_{j}^{-1}\frac{\partial h_{t}^{j}\left(x_{t}\right)}{\partial p_{x}^{t}}\frac{\partial h_{t}^{j}\left(x_{t}\right)}{\partial p_{y}^{t}}\\ \sum_{j=1}^{N}{\bar{R}}_{j}^{-1}\frac{\partial h_{t}^{j}\left(x_{t}\right)}{\partial p_{x}^{t}}\frac{\partial h_{t}^{j}\left(x_{t}\right)}{\partial p_{y}^{t}} & \sum_{j=1}^{N}{\bar{R}}_{j}^{-1}{\left(\frac{\partial h_{t}^{j}\left(x_{t}\right)}{\partial p_{y}^{t}}\right)}^{2} \end{array}\right) \end{array}$$

### Appendix A.2. FIM of Recursive Estimation

The FIM of recursive Bayesian estimation has been initially formulated by Petr *et al.* [36]. The formulation of the FIM, which indicates the estimation procedure of the conventional PFs, is similar to Equation (A4). The FIM of measurement data can be attained according to Equation (A11). Taking the prior information in $I_{P}\left(x_{t}\right)$ into account, the prior probability is the transition probability $p(x_{t}|x_{t-1})$. However, both $x_{t}$ and $x_{t-1}$ should be estimated during the process. Thus, the information about $x_{t-1}$ is also not accurate. Then, the estimated parameters for Fisher information is ${\left[x_{t},x_{t-1}\right]}^{T}$, and the related FIM including $I_{D}\left(x_{t},x_{t-1}\right)$ and $I_{P}\left(x_{t},x_{t-1}\right)$ should be reformulated.

For $I_{D}\left(x_{t}\right)$ computation, it is extended to be a 4×4 matrix, which is:

(A13) $$I_{D}\left(x_{t},x_{t-1}\right)=E\left\{\left[\frac{\partial \ln p(x_{t},z_{t})}{\partial x_{t}}\;\frac{\partial \ln p(x_{t},z_{t})}{\partial x_{t-1}}\right]{\left[\frac{\partial \ln p(x_{t},z_{t})}{\partial x_{t}}\;\frac{\partial \ln p(x_{t},z_{t})}{\partial x_{t-1}}\right]}^{T}\right\}$$

Since the current probability is independent of the previous state, $\frac{\partial \;\ln\;p(x_{t},z_{t})}{\partial x_{t-1}}=0$. Then, we obtain:

(A14) $$I_{D}\left(x_{t},x_{t-1}\right)=\left(\begin{array}{cc} I_{D}\left(x_{t}\right) & \\ & 0 \end{array}\right)$$

where 0 is a 2×2 zero matrix.

For the recursive process, $I(x_{t})$ is calculated recursively, which involves $I(x_{t-1})$ as the previous FIM. Then, $I_{P}\left(x_{t},x_{t-1}\right)$ is expressed as:

(A15) $$I_{P}\left(x_{t},x_{t-1}\right)=\left(\begin{array}{cc} Q_{t}^{-1} & \frac{\partial f_{t}\left(x_{t}\right)}{\partial x_{t-1}}Q_{t}^{-1}\\ {\frac{\partial f_{t}\left(x_{t}\right)}{\partial x_{t-1}}Q^{-1}}_{t}^{T} & \frac{\partial f_{t}\left(x_{t}\right)}{\partial x_{t-1}}Q_{t}^{-1}{\frac{\partial f_{t}\left(x_{t}\right)}{\partial x_{t-1}}}^{T}+I\left(x_{t-1}\right) \end{array}\right)$$

then, $I(x_{t},x_{t-1})$ is given:

(A16) $$I\left(x_{t},x_{t-1}\right)=I_{P}\left(x_{t},x_{t-1}\right)+I_{D}\left(x_{t},x_{t-1}\right)=\left(\begin{array}{cc} A & B\\ B^{T} & C+I(x_{t-1}) \end{array}\right)$$

where:

(A17) $$\begin{array}{cc} A & =I_{P}\left(x_{t}\right)+I_{D}\left(x_{t}\right)=Q_{t}^{-1}+I_{D}\left(x_{t}\right)\\ B & =-\frac{\partial f_{t}\left(x_{t}\right)}{\partial x_{t-1}}Q_{t}^{-1}\\ C & =\frac{\partial f_{t}\left(x_{t}\right)}{\partial x_{t-1}}Q_{t}^{-1}{\frac{\partial f_{t}\left(x_{t}\right)}{\partial x_{t-1}}}^{T} \end{array}$$

where $I(x_{t})$ is a part of $I({\left[x_{t},x_{t-1}\right]}^{T})$; A, B and C are parts of $I({\left[x_{t},x_{t-1}\right]}^{T})$, which represents the previous and current information [36]. Then, $I(x_{t})$ is given based on Schurcompletion:

(A18) $$I\left(x_{t}\right)=A-B{\left(I\left(x_{t-1}\right)+C\right)}^{-1}B^{T}$$
